# Performance on innate behaviour during early development as a function of stress level

**DOI:** 10.1038/s41598-017-08400-4

**Published:** 2017-08-10

**Authors:** Soojin Ryu, Rodrigo J. De Marco

**Affiliations:** 1 Developmental Neurobiology of Resilience, University Medical Center, Johannes Gutenberg University Mainz, Duesbergweg 6, 55128 Mainz, Germany; 2 Max Planck Institute for Medical Research, Jahnstr. 29, 69120 Heidelberg, Germany

## Abstract

What is the relationship between the level of acute stress and performance on innate behaviour? The diversity of innate behaviours and lack of sufficient data gathered under the same experimental conditions leave this question unresolved. While evidence points to an inverted-U shaped relationship between the level of acute stress and various measures of learning and memory function, it is unknown the extent to which such a non-linear function applies to performance on innate behaviour, which develops without example or practice under natural circumstances. The fundamental prediction of this view is that moderate stress levels will improve performance, while higher levels will not. Testing this proposition has been difficult because it entails an overall effect that must be invariant to the nature of the stressor, the behaviour under scrutiny and the stimulus that drives it. Here, we report new experimental results showing that developing zebrafish (*Danio rerio*) under moderate but not higher levels of stress improved their performance on instinctive activities driven by visual, hydrodynamic and thermal inputs. Our findings reveal, for the first time, the existence of an inverted-U shaped performance function according to stress level during early development in a series of innate behaviours.

## Introduction

Behaviour arises from a myriad of processes that guide the acquisition, processing and integration of sensory inputs, the connection between these inputs, past experiences and present physiological status, and the coordination of muscular activity by the prospective neural centres. By virtue of its complexity alone, behaviour is susceptible to disturbance by stress, whose consequences are assumed to combine and make up the state that is relevant to the effectiveness of a particular activity. There is little doubt that dysfunctional responses to stress can have deleterious consequences for health^1^, but stress can be beneficial too. In general, the relationship between the level of acute stress (i.e., glucocorticoid reactivity) and cognition is believed to follow an inverted U-shaped function^2, 3^. Although both components (i.e., ascending and descending) of this non-linear relationship have been difficult to pin down together, an inverted U-shape memory function according to stressor intensity has been confirmed under the same experimental conditions^4^. It remains unclear the extent to which the Yerkes-Dodson law applies to the relationship between acute stress and innate behaviour, which has high heritability and occurs inexorably without example or practice. Likewise, how widespread would such a relationship be and how early could it be detected? We address these issues in larval zebrafish using novel assays and the acute glucocorticoid response as a proxy for stress level. Zebrafish larvae are chosen because their hypothalamic-pituitary-interrenal (HPI) axis is homologous to the hypothalamic-pituitary-adrenal (HPA) axis^5^ and their small size allows for the continuous measurement of behaviour with full environmental control, including stressor onset. Also, they offer an excellent handle for the analysis of stress reactions and for non-invasive brain imaging and behavioural genetics^6–8^. The results below provide direct evidence for the existence of an inverted-U shaped relationship between stress level and performance on innate behaviour.

## Results

### Baseline, moderate and higher levels of stress

**Figure 1:**
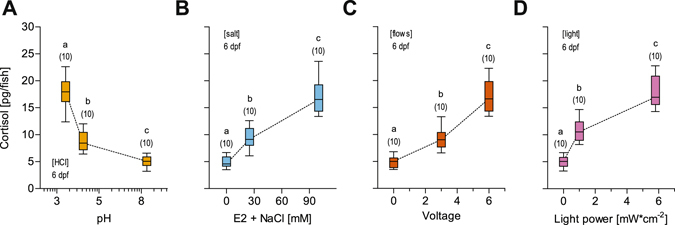
Stressors. (**A**–**D**) Whole-body cortisol in wild-type larvae (boxplots, whiskers: min to max, sample size in parentheses above the boxplots) as a function of stressor intensity: (**A**) pH drop (orange), (**B**) hyperosmotic medium (blue), (**C**) strong flows (vermilion) and (**D**) a squared pulse of light (purple). In all cases, stressor exposure increases whole-body cortisol in an intensity-dependent manner. Letters indicate results of Bonferroni’s tests (*p* < 0.001) after one-way ANOVAs.

We deployed two main approaches. Firstly, we exposed larvae to four different stressors of increasing intensity: pH drop, hyperosmotic medium, strong flows or light pulse (for details, see Methods). To compare the level of stress produced by these stimulations, we measured circulating levels of cortisol, a major stress hormone, directly after stressor exposure. The results confirmed that larvae can adjust whole-body cortisol to compensate for stressor intensity (Fig. 1, one-way ANOVA, pH drop: F(2,29) = 101.1, *p* < 0.0001, hyperosmotic medium: F(2,29) = 68.8, *p* < 0.0001, strong hydrodynamic flows: F(2,29) = 81.6, *p* < 0.0001, squared pulse of light: F(2,29) = 100.1, *p* < 0.0001, followed by *post hoc* comparisons). These measurements were used to establish baseline, moderate and higher levels of stress caused by single stressors, as well as similar levels caused by different stressors. In selecting the stimulations producing these levels, we avoided those causing maximum levels of stressor-mediated cortisol increase (not shown), linked to severe locomotion abnormalities and possibly other impairments. Baseline levels were those of control (unexposed) larvae from each of the four stimulation groups, which were equally handled, but the stressor was not present. Levels across these groups were similar (one-way ANOVA, F(3,39) = 0.02, *p* = 0.99) and gave cortisol means (±S.E.M.), in picograms (pg) per larva, of 5.01 (±0.34), 4.96 (±0.34), 4.90 (±0.35) and 5.00 (±0.34) for pH drop, hyperosmotic medium, strong flows and light pulse, respectively. Equally moderate levels (one-way ANOVA, F(3,39) = 1.9, *p* = 0.15) were reached via pH 4, NaCl 25 mM, 3 V and 1 mW*cm^−2^, with means (±S.E.M.) of 8.82 ± (0.60), 9.42 (±0.64), 9.24 (±0.62) and 10.83 (±0.66) pg/larva for each group, respectively, whereas equally higher levels (one-way ANOVA, F(3,39) = 0.2, *p* = 0.20) were reached via pH 3.3, NaCl 100 mM, 6 V and 5.8 mW*cm^−2^, with means (±S.E.M.) of 17.83 (±0.90), 17.07 (±1.05), 17.15 (±0.96) and 17.87 (±0.92) pg/larva for each group, respectively (see also Methods).

### Performance on innate behaviour as a function of stress level

Secondly, we determined the relationship between stress level and performance on innate behaviour. Larval zebrafish execute complex visually-guided behaviours, including a robust optomotor reflex^9^, i.e., spontaneous swimming in the direction of large-field displacements in the visual field. They use hydrodynamic sensing, which provides many species of fish with benefits ranging from object detection to sensing conspecifics^10^, and their response to rising temperature^6^ points to a fully functioning capacity for selecting best conditions in a thermal-gradient environment. Building on newly developed assays^6, 11–14^ and video tracking techniques, we assessed the larvae’s performance on a series of innate behaviours driven by visual, hydrodynamic and thermal inputs.

**Figure 2:**
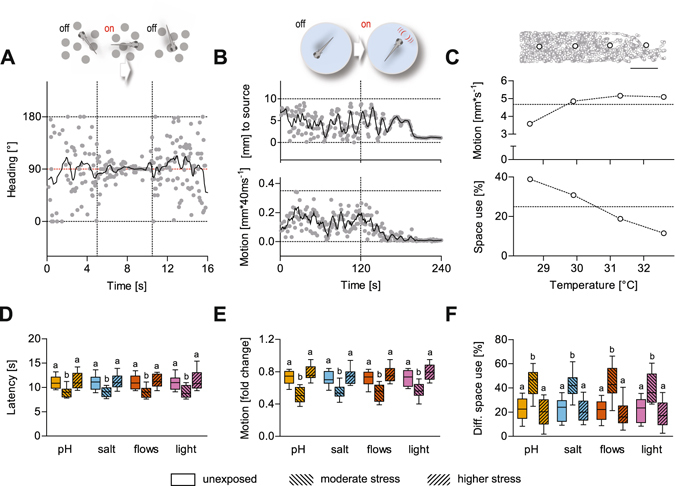
Innate behaviours and results. (**A**) Schematic (top) and representative trace (bottom) of an optomotor test depicting a larva’s heading, relative to the long axis of a rectangular swimming chamber, as a function of time. (**B**) Schematic showing the stimulation procedure (top) and representative traces of a larva’s distance to the stimulus source (middle) and swim velocity (bottom) before and after the onset of WMs (at 120 s). (**C**) Top, representative x-y coordinates (recorded every 40 ms over a 300 s period) of a freely behaving larva in a custom-made swimming chamber with a temperature gradient. White dots indicate the centre of each of the virtual quadrants. Scale bar, 5 mm. Middle, bottom, sample from a single larva, mean speed (middle) and proportion of time spent in each quadrant (bottom, space use, in %) over a 300 s period as a function of temperature. (**D**–**F**) Boxplots (whiskers: min to max) of latency (**D**), fold change in motion (**E**) and differential space use (**F**) values (from (**A**), (**B**) and (**C**), respectively) across groups of both control larvae (i.e., unexposed) and larvae with moderate and higher levels of stress caused by pH drop (orange), hyperosmotic medium (blue), strong flows (vermilion) and a squared pulse of light (purple) (see also Fig. 1 and ‘Results’). Letters indicate results of Bonferroni’s tests: (D), *p* < 0.01, (**E**,**F**), *p* < 0.001) after two-way ANOVAs. Sample size per group, 10.

To test responses to visual inputs, we used the optomotor reflex. In a first assay, we exposed larvae to visual field displacements caused by ventrally displayed dots^13^. A freely behaving larva swimming in a rectangular chamber displays no preferred heading if these dots remain stationary, but swims in the same direction of the dots if they move in parallel (Fig. 2A). As soon as the larva reaches any of the chamber’s far ends during a test, the dots begin to move in parallel towards the opposite end of the chamber. This causes the larva to align itself along the direction of the visual field displacement and swim together with the dots. The time interval in-between when the larva starts to swim with the dots and when it reaches the opposite end of the chamber (‘latency’) is taken as indicative of response strength: the lower the latency the higher the strength. In a second assay, we used highly controlled water motions (WMs) to test responses to minute hydrodynamic fields evoked at 5 Hz (Fig. 2B). Larvae respond to these non-stressful WMs with a pronounced reduction in locomotion combined with positive taxis towards the stimulus source^14^. To quantify their response, we measured integrals of distance swum against time for equal periods before and during WMs, and used ‘fold change in motion’ as indicative of a larva’s response to the stimuli: the higher the locomotion reduction the higher the response. Finally, in a third assay, we studied a larva’s ability to avoid above preferred (i.e., raising) water temperature. For this we monitored the percentage of time that single larvae spent in each -virtual- quadrant of an elongated chamber offering a stable temperature gradient (Fig. 2C). To measure response strength, we calculated ‘differential space use (in %)’ for each larva, as the difference between the percentage of time spent in the low and the high temperature quadrant.

**Figure 3:**
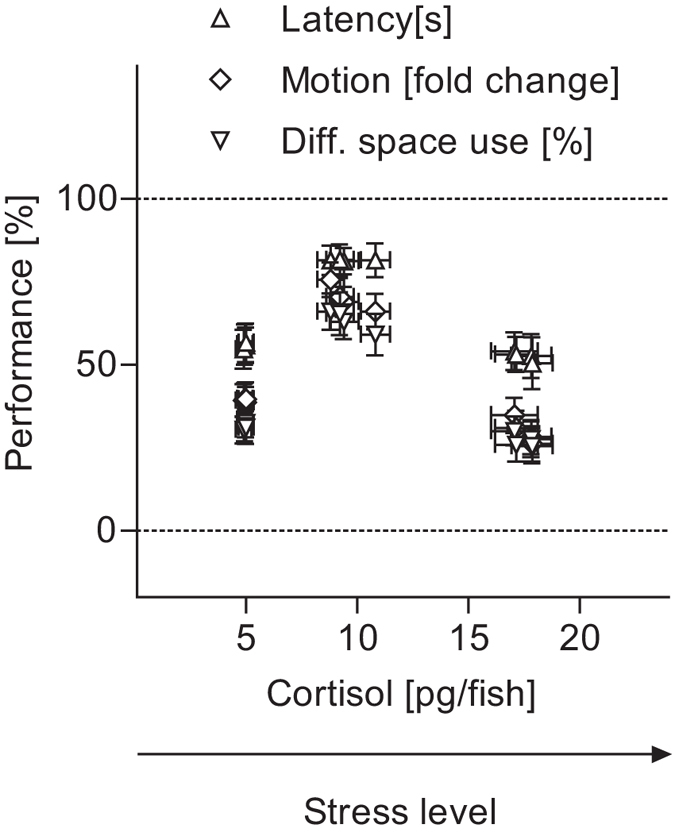
Performance as a function of stress level. Relative performance values (mean ± s.e.m., in %, as functions of min and max values) against whole-body cortisol for the data in Fig. 2D–F. Sample size per group, 10.

To assess the effect of stress on these behaviours, we compared ‘latency’, ‘motion fold change’ and ‘differential space use’ values across groups of larvae with baseline, moderate and higher levels of whole-body cortisol. In all three cases, the results showed that moderate but not higher stress levels improved performance (Fig. 2D–F, two-way ANOVA, D, level: F(2,108) = 31.6, *p* < 0.0001, type: F(3,108) = 0.02, *p* = 0.99, level × type: F(6,108) = 0.04, *p* = 0.99, E, level: F(2,108) = 68.5, *p* < 0.0001, type: F(3,108) = 0.37, *p* = 0.77, level × type: F(6,108) = 0.40, *p* = 0.88, F, level: F(2,108) = 59.2, *p* < 0.0001, type: F(3,108) = 0.26, *p* = 0.85, level × type: F(6,108) = 0.15, *p* = 0.99, followed by *post hoc* comparisons). To complement these assessments, we plotted relative performance values (in %), as functions of the minimum and maximum values from each of the three assays, against the level of whole-body cortisol (Fig. 3). Figure 3 shows that, upon moderate stress, performance increased on average by a factor of 1.5, 1.8 and 2 in responses driven by visual, hydrodynamic and thermal inputs, respectively, in agreement with previous data^6^.

## Discussion

In zebrafish, basal levels of whole-body cortisol and expression levels of genes responsible for corticosteroid synthesis and signalling increase drastically around the time of hatching, revealing an HPI axis that matures early in development^15–17^. The larvae’s neurosecretory preoptic-hypothalamic area (NPO) is homologous to the mammalian hypothalamic paraventricular nucleus, innervation of the pituitary by NPO cells is established by 3 days post fertilization, and ablation and activation of cortisol-producing interrenal cells reduces and increases cortisol upon stress, respectively^18–20^. Two-photon calcium imaging on intact larvae shows that the activity of NPO-cells producing corticotropin-releasing hormone, the primary stimulator of the HPA axis, is highly synchronized and co-varies with stressor intensity^21^. Interactions of GRs and serotonin signalling are present too^22^, and larvae show elaborate stress reactions^6, 21, 23–25^, including a reversible suppression of the feeding drive^13^, a common phenomenon among vertebrates also found in adult teleosts^26, 27^. Furthermore, evidence also shows that serotonergic neurons in the dorsal raphe can modulate sensory responsiveness^28^ as well as light/dark preference^29^, depending on their activity level. The present data set shows an inverted-U shaped relationship between the level of acute stress and a larva’s performance on innate behaviour, complementing recent findings of enhanced stimulus responsiveness after stressor exposure^6^ and a recovery of the feeding drive that is faster under intermediate but not higher levels of salt stress^13^.

The results are exceptional in identifying such a non-linear relationship so early in life. A key question now emerging is how it relates to survival in a species facing greater mortality during early development. For developing zebrafish, increasing performance under moderate stress levels may have advantages. Such ability may help larvae to better cope with environmental variability and predation risk, lessening a strong pressure for survival. Another emergent question relates to the study of behaviour, resilience and fitness as a function of maturation. Ontogeny is concerned with the roles of genetics and maturation in shaping an animal’s life history. All behaviours are influenced by an animal’s genetic background and the environments that exist during development. The extent to which the two influences determine the outcome varies from species to species, and from activity to activity within a species. It is generally assumed that the acute response of the HPA axis can be beneficial, and that its positive effects may be linked to epigenetic programming caused by coping with stress^30^. There is, however, a complete lack of evidence linking early activation patterns of the HPA axis to direct measures of performance, resilience and fitness^31^. In larval zebrafish, all three elements of the HPI axis can be genetically visualized and manipulated^6^ and inbred populations of larvae can be used to link early environments to HPA axis activation patterns and measures of performance, survival and reproductive outcome. Our findings suggest the relationship between acute stress and behavioural performance following an inverted-U curve as a widespread phenomenon among vertebrates. Both environmental and resource uncertainty under great competition may have set the circumstances that selected for it. Elucidation of the mechanisms underlying such relationship is central to the analysis of phenotypic adaptation.

## Methods

### Zebrafish husbandry and handling

Zebrafish breeding and maintenance were performed under standard conditions^32^. Wild-type embryos (cross of AB and TL strains, AB/TL) were collected in the morning and raised on a 12:12 light/dark cycle in E2 medium at 28 °C. All experiments were carried out with larvae at 6 days post fertilization (d.p.f.). Tests were performed between 09:00 hours and 18:00 hours, with different experimental groups intermixed throughout the day. Zebrafish experimental procedures were performed according to the guidelines of the German animal welfare law and approved by the local government (Regierungspräsidium Karlsruhe; G-29/12).

### Stressors

Groups of thirty larvae in 30 mm Petri dishes were exposed to known stress protocols^6, 11–13, 21^, each based on one of four different stimulations: HCl (pH drop), NaCl (hyperosmotic medium), strong hydrodynamic flows or a squared pulse of light. They were then used for cortisol measurement or transferred to custom-made swimming chambers for behavioural testing. *pH drop*: larvae were incubated for 3 min in steady-state E2 medium (unexposed) or E2+ varying concentrations of hydrochloric acid (Merck, #109063) at 28 °C under white-light illumination. They were then washed three times with E2 medium and kept in a small container for cortisol detection (6 min later), or transferred to a custom made swimming chamber for behavioural testing (12 min later). The wash and transfer period lasted 3 min (±10 s) and was performed at room temperature. *Hyperosmotic medium*: larvae were incubated for 10 min in steady state E2 medium (unexposed) or E2+ varying concentrations of NaCl (Merck, #106404) at 28 °C under white light illumination. They were also washed three times with E2 medium and kept in a small container for immediate cortisol detection, or transferred to a swimming chamber for behavioural testing (5 min later). As before (i.e., pH drop), the wash and transfer period took 3 min (±10 s) and was performed at room temperature. *Strong hydrodynamic flows*: larvae were presented with water motions caused by rapid lateral displacements of a rigid silica capillary tube (Polymicro Technologies, AZ, 360 µm OD, Optronis GmbH; Kehl, Germany) fixed to a multilayer piezo bender actuator (PICMA^®^ PL140.10, Physik Instrumente (PI) GmbH and Co. KG; Karlsruhe, Germany). The actuator had an operating voltage of 0–60 V, a maximum displacement of ±1000 µm, and an unloaded resonant frequency of 160 Hz. The bender was connected to a dual-piezo-amplifier (maximum voltage: 10 V), a pulse generator and a TTL control box (USB-IO box, Noldus Information Technology, Wageningen, The Netherlands) allowing for computer control. The tip of the silica capillary tube was submerged (2 mm) at the centre of a 30 mm Petri dish, half filled (1.8 ml) with E2 medium (orientation relative to water surface: 90°). The voltage applied to the bender (V_act_) determined the speed of the capillary’s lateral displacements, or stimulus strength (in % relative to maximum voltage). Groups of 30 larvae were exposed to 6 stimulation units delivered with an inter-stimulation-interval of 250 ms. Each unit consisted of 99 repetitions of 40 ms lateral displacements. We used a V_act_ of 3, 4.5 or 6 V. Stimulations were carried out under white illumination at 28 °C. After stimulation, larvae were kept in Petri dishes for cortisol measurement (9.5 min later), or transferred to a swimming chamber for behavioural testing, where they remained without perturbation for 10 min before recordings. *Light pulse*: larvae were dark-adapted for 3 min and then exposed to a 180 s squared pulse of flashing blue light of varying light power, as described elsewhere. Custom-made drivers, amplifiers, pulse generators and the USB-IO box (see above) allowed computer control of the squared pulse of light, delivered through a custom-made array of LEDs placed inside a light-proof enclosure (see below). The LEDs were positioned at a fixed distance above the Petri dish. The incident angle of the LEDs allowed for homogeneous illumination of the dish. Each squared pulse of light consisted of 100 ms flashes delivered at 5 Hz. Light power was measured through a hand-held light power meter (Newport Corp, Irvine, CA, USA). After light exposure, larvae were kept in the dish for cortisol measurement (6 min later), or transferred to a swimming chamber for behavioural testing, where they remained without perturbation for 12 min before recordings.

### Independent sampling

Cortisol and behavioural measurements were made on different groups of equally treated larvae and therefore constitute fully independent samples. For the behavioural measurements, each replicate involved a single larva. Yet, these individual measurements were made on larvae that had also been kept in wells containing a total of thirty larvae per well. Thus, the number of single larvae matched the number of independent wells. In this manner, the density of larvae per well during stressor exposure remained a constant factor for both the cortisol and behavioural measurements. In sum, for each cortisol measurement, all thirty larvae in a well were used, whereas each behavioural measurement involved only one larva - the remaining twenty-nine larvae in the well were used elsewhere outside the study. In sum, each replication was fully independent from the others thus avoiding pseudo-replication.

### Whole-body cortisol

Groups of thirty larvae were immobilized in ice water after being exposed to HCl, NaCl, strong flows or the light pulse. Unexposed larvae (control samples) were collected after equal handling, omitting stressor exposure. Samples were then frozen in an ethanol/dry-ice bath and stored at −20 °C for subsequent extraction. Each replicate consisted of a well with 30 larvae. Cortisol extraction and detection were carried out using a home-made cortisol ELISA protocol, as described elsewhere^11^.

### Test overview

Behavioural experiments using visual, hydrodynamic and thermal inputs were conducted under both white and infrared illumination, delivered through a custom-made array of white- and infrared-LEDs mounted inside a light-proof enclosure. Tests involving hydrodynamic and thermal inputs were conducted under infrared light only. The complete setup was placed on a vibration-free platform (Newport Corp, Irvine, CA, USA). Larvae were imaged at 25 frames s^*−*1^ (camera: ICD-49E B/W, Ikegami Tsushinki Co, Ltd, Japan) with a lens (TV Lens, Computer VARI FOCAL H3Z4512 CS-IR, CBC; Commak, NY, USA) positioned above rectangular (visual and thermal inputs) or cylindrical (hydrodynamic input) custom made swimming chambers. EthoVision XT 7 software (Noldus Information Technology) was used to monitor the movements of individually swimming larvae. Motion values were expressed as distance swum every second (mm per 1 s) or 40 ms (mm per 40 ms). All experiments involved single larvae moving freely within the swimming chamber. Before tests, each larva was given an initial time period of several minutes to adapt to the chamber’s conditions. Experiments were conducted at 28 ± 1 °C, unless otherwise stated. A thermocouple (npi electronics GmbH; Tamm, Germany) connected to a temperature control system (PTC 20, npi electronics GmbH; Tamm, Germany; Exos-2 V2 liquid cooling system, Koolance; Auburn, WA, USA) monitored the temperature inside the swimming chamber. All the experiments were performed in a blind fashion as to group identity. Control animals for each group were handled in the same fashion, but omitting stressor presentation.

### Visual input

Control and treated larvae were placed individually in a rectangular chamber (length: 40 mm, width: 20 mm, height: 10 mm) filled with 3 ml of E2 medium and mounted above a horizontally oriented computer screen. The screen displayed visual stimuli created via an algorithm written in MATLAB 2009b (MathWorks, Inc.; Natick, MA, USA). Visual stimuli consisted of grey dots (displayed against a white background offering a light power of 0.5 mW*cm^−2^) of fixed contrast, diameter (in degrees), velocity (in degrees s^−1^) and number, as previously defined^13^. In each session, a larva was first presented with stationary dots and allowed to swim freely for 60 s. Next, once the test started, as soon as the larva reached any of the chamber’s far ends, the dots automatically began to move in parallel towards the opposite end of the chamber. As a result, due to the optomotor reflex, the larva would align itself along the direction of the large visual field displacement and begin to swim consistently with the moving dots towards the opposite far end of the chamber. To quantify response strength, we measured ‘latency’, in seconds, as the time interval in-between when the larva started to move with the dots and when it reached the opposite end of the chamber.

### Hydrodynamic input

Computer control of minute water motions was achieved through custom-made drivers, amplifiers, pulse generators and the USB-IO box, as described below; for details, see also ref. 14. The cylindrical swimming chamber (internal diameter: 10 mm, height: 10 mm) had a transparent bottom and two opposite overtures, inlet and outlet (width: 2.5 mm, height: 400 μm), allowing the medium (E2) to flow at 200 μl min^−1^ with the aid of a peristaltic pump (IPC Ismatec, IDEX Health and Science GmbH, Wertheim, Germany). The chamber also had two cylindrical side channels (internal diameter: 400 μm) opposite to each other opening 200 μm above the transparent glass bottom, with their longest axis oriented at an angle of 30° relative to horizontal. One such channel held the thermocouple monitoring the temperature inside the chamber, while the other allowed passage of the end of a rigid silica capillary tube, or stimulus source (outer diameter: 350 μm, full length: 25 mm, Polymicro Technologies), submerged ~400 μm into the chamber’s inner medium (depth: 5 mm). The opposite end of the capillary tube was fixed to a multilayer bender actuator (PICMA PL140.10, Physik Instrumente (PI) GmbH + Co. KG, Karlsruhe, Germany) with an operating voltage of 0–60 V, a maximum displacement of ±1,000 μm and an unloaded resonant frequency of 160 Hz. The bender, coupled to a pulse generator, a dual piezo amplifier and a TTL control system, produced unidirectional lateral displacements (of 50 μm and controllable speed) of the capillary’s submerged end, creating minute, non-stressful WMs within the chamber. The input voltage applied to the actuator (0.5 V) determined the speed of the capillary’s lateral displacements. Single larvae in the swimming chamber were video-recorded for 120 s under infrared light and constant temperature. They were then presented with 1 ms lateral displacements of the silica capillary tube delivered at 1 Hz (input voltage: 0.5 V) for 120 s. Motion before and during stimulation was calculated using the integrals of motion over 120 s.

### Thermal input

We used a custom-made, rectangular test chamber (length: 24 mm, width: 5 mm, height: 10 mm) with two vertically-oriented walls (width: 5 mm, height: 10 mm, thickness: 500 µm) at its opposite ends. These two end-walls had contiguous rows of equidistant 100 µm openings allowing the E2 medium to diffuse. On each side of the chamber, beyond the end-walls, two opposite overtures (inlet and outlet, diameter: 2.5 mm) allowed the medium to flow at 150 μl min^−1^ with the aid of the pump (IPC Ismatec, IDEX Health and Science GmbH, Wertheim, Germany). In between the end-walls and the overtures (inlet and outlet), thermocouples (TS200, npi electronics GmbH, Tamm, Germany) monitored the temperature of the flowing medium. One such thermocouple provided feedback to a control system (PTC 20, npi electronics GmbH; Exos-2 V2 liquid cooling system, Koolance, Auburn, WA, USA) that either kept the flowing medium at 28 °C (±0.1 °C) or increased its temperature in a highly controlled manner until a stable temperature gradient was established inside the chamber and confirmed via independent measurements. In each recording session, a single larva, which had been kept for 10 minutes at 28 °C (±0.1 °C) in a second, parallel chamber offering exactly the same medium and flow conditions, was moved into the test chamber and video-recorded for 300 s under infrared light. To assess avoidance of above preferred (i.e., raising) water temperature (28 °C), the chamber was divided in four virtual quadrants equal in size and the percentage of time spent in each of these quadrants over the entire recording session was used as a measure of space use. The resulting ‘differential space use’ values (in %) were then calculated as the difference between the percentage of time spent in the low and high temperature quadrant for each larva.

### Statistics

All data are shown as boxplots (median and whiskers: min to max) ﻿except indicated otherwise. We used a random experimental design and ANOVAs for multiple group comparisons (followed by Bonferroni’s *post hoc* tests). Normality was tested using Kolmogorov–Smirnov, Shapiro–Wilk and D’Agostino tests. Analyses were made with MS-Excel (Microsoft Corp; Redmond, WA, USA) and Prism 5 (Graphpad Software Inc, San Diego, CA, USA).

### Data accessibility

The data that support the findings of this study are available from the authors on request.
